# Catecholamine Metabolism in Paraganglioma and Pheochromocytoma: Similar Tumors in Different Sites?

**DOI:** 10.1371/journal.pone.0125426

**Published:** 2015-05-06

**Authors:** Eric Grouzmann, Oliver Tschopp, Frédéric Triponez, Maurice Matter, Stefan Bilz, Michael Brändle, Tilman Drechser, Sarah Sigrist, Henryk Zulewski, Christoph Henzen, Stefan Fischli, Karim Abid

**Affiliations:** 1 Service of Biomedicine, Catecholamine and Peptides Laboratory, Centre Hospitalier Universitaire Vaudois (CHUV), Lausanne, Switzerland; 2 Division of Endocrinology, Diabetes and Metabolism, University Hospital Zurich, Zurich, Switzerland; 3 Department of Thoracic and Endocrine Surgery, University Hospital Geneva (HUG), Geneva, Switzerland; 4 Division of Visceral Surgery, Centre Hospitalier Universitaire Vaudois (CHUV), Lausanne, Switzerland; 5 Clinic for Endocrinology, Diabetes, Bone disease and Metabolism, KantonsSpital St. Gallen, St. Gallen, Switzerland; 6 Division for Endocrinology, Diabetes and Metabolism, University Hospital Basel, Basel, Switzerland; 7 Division of Endocrinology and Diabetes, KantonsSpital Luzern, Luzern, Switzerland; UPR 3212 CNRS -Université de Strasbourg, FRANCE

## Abstract

Pheochromocytoma (PHEO) and paraganglioma (PGL) are catecholamine-producing neuroendocrine tumors that arise respectively inside or outside the adrenal medulla. Several reports have shown that adrenal glucocorticoids (GC) play an important regulatory role on the genes encoding the main enzymes involved in catecholamine (CAT) synthesis *i.e. tyrosine hydroxylase (TH)*, *dopamine β-hydroxylase (DBH) and phenylethanolamine N-methyltransferase (PNMT)*. To assess the influence of tumor location on CAT metabolism, 66 tissue samples (53 PHEO, 13 PGL) and 73 plasma samples (50 PHEO, 23 PGL) were studied. Western blot and qPCR were performed for TH, DBH and PNMT expression. We found a significantly lower intra-tumoral concentration of CAT and metanephrines (MNs) in PGL along with a downregulation of TH and PNMT at both mRNA and protein level compared with PHEO. However, when PHEO were partitioned into noradrenergic (NorAd) and mixed tumors based on an intra-tumoral CAT ratio (NE/E >90%), PGL and NorAd PHEO sustained similar TH, DBH and PNMT gene and protein expression. CAT concentration and composition were also similar between NorAd PHEO and PGL, excluding the use of CAT or MNs to discriminate between PGL and PHEO on the basis of biochemical tests. We observed an increase of TH mRNA concentration without correlation with TH protein expression in primary cell culture of PHEO and PGL incubated with dexamethasone during 24 hours; no changes were monitored for PNMT and DBH at both mRNA and protein level in PHEO and PGL. Altogether, these results indicate that long term CAT synthesis is not driven by the close environment where the tumor develops and suggest that GC alone is not sufficient to regulate CAT synthesis pathway in PHEO/PGL.

## Introduction

Pheochromocytoma (PHEO) and paraganglioma (PGL) are tumors arising from chromaffin cells that derive from the embryonic neural crest. The majority of PHEO/PGL cases are known as sporadic tumors while mutations in genes including *VHL* (von Hippel-Lindau), *RET* (Multiple Endocrine Neoplasia type 2), *NF1* (Neurofibromatosis type 1), *SDH* (Succinate Dehydrogenase subunits A, B, C and D) and cofactor *SDHAF2*, *MAX* (MYC associated factor X), *HIF2A* (hypoxia-inducible factor 2A), *FH* (fumarate hydratase) and *TMEM127* (transmembrane protein 127) account for approximately 40% of tumors [1]. PHEO are located within the adrenal medulla and PGL (also formerly known as extraadrenal pheochromocytoma) are found in the sympathetic and parasympathetic ganglia [2]. PGL are more likely to develop metastasis with an incidence rate of approximately 10% of total PHEO/PGL cases. Both types of tumors produce and usually secrete larger amounts of catecholamine (CAT) than the adrenal medulla, due to an up-regulation of tyrosine hydroxylase (TH; EC 1.14.16.2) and dopamine β-hydroxylase (DBH, EC 1.14.17.1) the main enzymes responsible for CAT synthesis. In chromaffin cells and pheochromocytes, norepinephrine (NE) and epinephrine (E) are stored in vesicles where they sustain a passive leakage into the cytoplasm before being recaptured in the vesicle pool. Adrenal medulla is by far the most important site of E production in the body since phenylethanolamine N-methyltransferase (PNMT; EC 2.1.1.), the enzyme that transforms NE into E, is largely restricted to this tissue and absent from sympathetic nerves that only produce NE [3–5]. PNMT appears to be down regulated in a large number of PHEO, except in E-secreting tumors. While in adrenal medulla approximately 80% of CAT consist of E, in many PHEO, NE largely prevails over E in terms of production. PNMT downregulation has been attributed to dysregulation in hormone and transcription factor concentration that include glucocorticoids (GC) carried from adjacent cortical cells to medulla by the adrenal portal system [6–12].

In the context of the PHEO-exclusion diagnosis tests performed in our laboratory, we have noticed lower E and metanephrine (MN) plasma concentrations in patients affected by a PGL compared to PHEO. This prompted us to study the underlying molecular mechanisms responsible for the decrease of CAT and especially E synthesis in PGL compared to PHEO. CAT metabolism, TH, DBH and PNMT expression at both mRNA and protein levels were assessed in both kinds of tumors. Due to high heterogeneity of PHEO regarding CAT production and metabolism, we further arbitrarily divided PHEO into two subgroups; mixed PHEO (Mix) and noradrenergic PHEO (NorAd), arbitrarily based on an intratumoral CAT ratio of NE/E >90% [13].

## Material and Methods

### Subjects and samples

**Fig 1:**
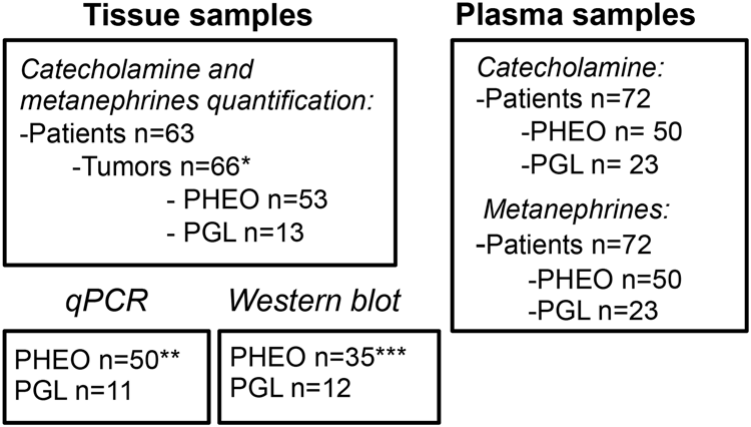
Flowchart of the patients and tumor samples included in the study. For CAT and MNs quantification in tissue 63 patients were enrolled and 66 tumors were collected (two patients had bilateral tumors (P21 and P43) and one had both a PHEO and a PGL (P25)*, see also Table 1. Tumors consisted of 53 PHEO and 13 PGL. Quantification of CAT in plasma was performed on 72 patients from 50 PHEO and 23 PGL patients, (including P25 with both a PHEO and a PGL). The plasma CAT analysis was performed on the same patient recruited for tissue CAT and MNs study with the following exceptions: two plasma were not available (P14 and P27) and two tumors were bilaterally excised at the same time (P43 and P44), finally, 10 additional plasma from PGL patients were added in the study without tissue sample available. For MNs quantification in plasma, sample P62 was not available (instead of P27 for CAT). Three tissue samples for PHEO and two for PGL were stored in perchloric acid, not compatible for mRNA extraction for qPCR assay**. 15 PHEO tissue sample were no longer available for WB assays***.

Fresh tumor specimens were obtained surgically between 2006 and 2014 in 11 different hospitals and clinics in Switzerland, from 63 patients with histologically confirmed PHEO (13 PGL and 53 PHEO, 2 patients with bilateral adrenalectomy: (P21/P28 and P43/P44) and one patient with a PHEO and a simultaneous PGL without signs of malignancy (P25 and P26), (Table 1). The 27 women and 25 men with PHEO had a mean age of 49 ± 16.9 years (range 12 to 78). For PGL the cohort consisted of 8 women and 5 men, mean age of 40+/-18.3 years (range 13–72). Blood samples were collected after 15 min of supine rest. Plasma CAT and MNs were measured in the same patients having a PHEO, except for two patients whose CAT values in plasma were not available (P14 and P27), and one additional patient affected by a men2a syndrome that underwent double adrenalectomy at the same time (P43). For PGL there were 10 additional plasma values on top of the 13 for which tissue biopsies were available, without corresponding intra-tumoral values (tissue not collected) (Fig 1). All tumors/plasma tested for metabolites/mRNA or protein quantification were included in the results reported here. Among the 63 patients; 47 were affected by a sporadic tumor and 16 by genetic associated tumors (6 had mutations on *RET* gene, 4 on *SDHB*, 3 on *NF1* and 3 on *VHL*. 18 patients out of 47 sporadic PHEO cases were investigated for genetic disease and found to be devoid of SDHA, SDHB, SDHC, SDHD, RET, VHL or MAX mutations (Table 1 and S1 Table). Identified mutations are reported in supporting information (S1 Table). The decision to perform genetic analysis was based on patient’s age, suspicion of malignancy and germline mutation and consent in all cases. There is currently no guidelines in Switzerland to perform systematic genetic analysis on PHEO/PGL. Malignancy was confirmed when metastasis were shown to develop in non-neuroendocrine tissue. Point mutations were screened by automated Sanger sequencing and detection of intragenic deletions/duplication using multiplex ligation-dependent probe amplification (MLPA) The study was reviewed and approved by the Ethical Committee of the Clinical Research of the Biology and Medecine Faculty of the University of Lausanne, Lausanne, Switzerland. Protocol number 95/04, “study of the secretion of catecholamine and metabolites from human adrenal gland and pheochromocytoma". All patients provided their written consent to participate in this study and the ethic committee approved the consent procedure. Parents gave the written consent for any minors included in this study.

**Table 1:** Patients and tumors characteristics.

Patient code	Age	Gender	Localisation	Malignancy	Diameter cm	Weigth g	PGL	Genetic	Mix-/NorAd-tumor	Hypertension	NE nmol/L	E nmol/L	Free NMN nmol/L	Free MN nmol/L
P01	21	F	L	-	3.5	nd	-	men2a	Mix-	-	2.6	0.1	3.1	1.3
P02	62	F	L	-	4.5	38	-	NT	Mix-	+	135.4	57.5	12.4	13.7
P03	54	F	R	-	6x6x3	nd	-	NT	NorAd	+	7.8	0.3	28.9	0.5
P04	44	M	R	-	11	122	-	Sp*	Mix-	+	216.9	28.5	23.2	6.4
P05	67	M	R	-	3.5x3x3.5	nd	-	NT	NorAd	+	59.2	2.5	8.0	1.5
P06	68	F	L	-	16	1070	-	Sp**	NorAd	+	14.0	0.4	65.0	0.4
P07	72	F	abd	-	8	100	+	NT	Mix-	+	1.2	0.6	3.4	5.1
P08	78	F	R	-	3.7	nd	-	Sp**	Mix-	+	0.8	0.8	0.6	2.3
P09	40	M	L	-	6x5.8x5.6	nd	-	NT	Mix-	+	49.1	5.1	18.4	4.1
P10	58	M	L	+	10	nd	-	Sp*	NorAd	+	102.4	0.4	83.6	0.6
P11	25	M	abd	-	10x6x3	115	+	SDHB	NorAd	+	48.3	0.0	6.7	0.5
P12	53	M	abd	-	4.5	20	+	Sp*	Mix-	+	45.8	0.1	3.6	0.1
P13	64	M	L	-	4	nd	-	NT	Mix-	+	5.0	2.6	3.6	4.3
P14	48	F	L	-	3	16	-	NT	Mix-	+	nd	nd	nd	nd
P15	35	M	L	-	4	47	-	Sp*	NorAd	+	20.1	0.2	7.6	0.2
P16	20	F	abd	+	7.5	nd	+	SDHB	NorAd	+	60.8	0.1	7.2	0.1
P17	20	M	L	-	2.5	15	-	men2a	Mix-	+	1.5	0.1	0.6	1.0
P18	43	F	L	-	3	92	-	men2a	Mix-	-	1.4	0.2	2.3	12.8
P19	22	M	R	-	3x2x2	38	-	Sp*	NorAd	+	12.3	0.4	4.0	0.2
P20	30	F	R	-	9.5	128	-	NF1	NorAd	+	63.6	1.2	37.8	2.3
P21	41	F	R	-	3	20	-	men2a	Mix-	-	4.7	1.4	2.3	4.3
P22	33	M	1	+	7x5x3	nd	+	Sp^1^	Mix-	+	56.9	0.1	11.4	0.2
P23	44	F	L	-	14.5	298	-	NT	NorAd	+	24.5	0.2	43.1	0.1
P24	64	F	L	-	3	nd	-	NT	Mix-	-	3.1	0.6	2.8	2.1
P25	19	M	L	-	2.5	23	-	Sp**	NorAd	-	6.88	0.25	2.1	0.2
P26	19	M	abd	-	1	nd	+	Sp**	NorAd	-	6.9	0.25	2.05	0.2
P27	53	M	L	-	1.5	32	-	NT	NorAd	+	nd	nd	1.7	0.1
P28	43	F	L	-	7	37	-	men2a	Mix-	-	7.0	1.1	2.7	1.9
P29	66	F	L	-	8x8x7	209	-	NT	NorAd	+	159.0	0.6	87.6	0.7
P30	42	F	2	-	5.5	nd	+	Sp^2^	NorAd	-	6.7	0.2	3.2	0.2
P31	66	M	R	-	7	49	-	NT	Mix-	+	3.4	0.5	4.5	1.2
P32	25	M	3	-	6.5x4.5x4.2	nd	+	NT	Mix-	nd	2.1	0.1	0.4	0.1
P33	68	F	L	-	4.5	nd	-	NT	Mix-	+	40.1	1.2	12.5	1.4
P34	63	M	L	-	10	nd	-	Sp*	Mix-	+	23.3	4.9	29.1	45.9
P35	50	F	4	-	5.7	nd	+	Sp	Mix-	+	19.4	0.2	19.0	0.4
P36	64	F	R	-	4.5	14	-	NT	NorAd	+	5.6	0.2	9.4	0.3
P37	54	M	R	-	5.5x5.7	69	-	NT	NorAd	-	14.3	0.3	5.1	0.2
P38	13	M	R	-	nd	nd	-	Sp^3^	NorAd	nd	44.6	0.1	7.3	0.2
P39	12	M	L	-	3x2.5x1.7	nd	-	VHL	NorAd	+	1.9	0.3	4.1	0.2
P40	47	M	L	-	3	22	-	NF1	Mix-	-	3.4	0.5	2.6	1.6
P41	42	F	L	-	4x4x3	nd	-	NT	Mix-	+	3.0	0.5	3.7	1.7
P42	51	F	5	+	8x4.5	nd	+	SDHB	Mix-	+	14.9	0.4	3.3	0.2
P43	33	M	L	-	4	nd	-	men2a	Mix-	+	3.5	1.3	21.3	12.2
P44	33	M	R	-	8	nd	-	men2a	Mix-	+	3.5	1.3	21.3	12.2
P45	66	F	L	-	4	nd	-	NT	Mix-	+	4.1	0.9	2.7	1.9
P46	72	F	L	-	3.2	nd	-	NT	NorAd	nd	71.9	1.0	16.1	0.8
P47	13	M	liver	-	nd	nd	+	Sp^2^	NorAd	nd	92.0	0.5	10.5	0.3
P48	59	M	L	-	1.4	nd	-	NT	Mix-	+	3.5	0.3	0.5	0.5
P49	57	F	cervical	+	3x3.5x4	nd	+	SDHB	NorAd	+	164.0	0.2	101.0	0.2
P50	72	M	L	-	4.5x3.8x2.8	58	-	NT	Mix-	+	4.4	3.1	4.3	3.8
P51	68	F	L	-	3.5x3.3	nd	-	NT	Mix-	+	37.2	4.7	11.0	5.5
P52	40	M	R	-	4x3x3.4	nd	-	Sp^4^	Mix-	+	3.3	0.4	1.3	3.5
P53	49	F	R	-	4	nd	-	NT	Mix-	+	7.8	9.5	3.2	9.4
P54	33	M	R	-	5.5x3.3x2.7	49	-	Sp*	NorAd	-	2.6	0.1	6.6	0.2
P55	29	F	L	-	4.2x3.9x6	nd	-	NT	Mix-	-	0.4	2.0	0.4	0.2
P56	52	F	R	-	2x2.7x2.5	nd	-	NT	NorAd	+	7.8	0.0	3.5	0.1
P57	42	M	L	-	1.7x.1.5x1.3	nd	-	VHL	NorAd	-	2.0	0.2	0.8	0.2
P58	70	M	R	-	4.5x4.5x2.9	nd	-	Sp*	Mix-	+	7.0	2.2	4.0	1.6
P59	55	F	L	-	3.1	nd	-	Sp^5^	NorAd	+	5.8	0.6	3.1	0.3
P60	52	M	R	-	4.5x5.5	nd	-	NF1	NorAd	+	28.4	1.6	8.3	1.2
P61	64	M	L	-	7.5x5.8x5.8	86	-	NT	Mix-	+	3.1	6.1	1.3	14.2
P62	37	M	L	-	6.1	nd	-	men2a	Mix-	+	22.9	4.2	nd	nd
P63	27	F	L	-	9	nd	-	VHL	NorAd	-	5.1	0.1	17.1	0.1
P64	36	M	R	-	2x1.9x1.8	nd	-	NT	NorAd	+	7.0	0.7	1.7	0.3
P66	59	M	L	-	4.6x3.3x3.4	33	-	NT	Mix-	+	3.2	1.9	5.4	5.4
P67	57	F	abd	-	1.6x.1.9	nd	+	NT	NorAd	+	4.6	0.6	2.5	0.3

Localisation: PHEO were found either in the left (L) or right (R) adrenal gland. PGL were excised from the following location: abd: abdominal; 1: paraortal right; 2: paraortal left; 3: Glomus vagal right; 4: retroperitonal left, paraortal; 5: abdominal, paraortal. NT: non-tested, Sp*: sporadic, RET, SDHB, -D and VHL negative for mutation

Sp**: RET and SDHB negative. SP^1^: SDHA, -B, -C, -D, MAX, VHL negative. SP^2^: SDHB, -D, RET, VHL, Men1 negative, SP^3^: SDHB, -C, -D, RET, negative, SP^4^: VHL negative, SP^5^: SDHB, -C, -D, Men1, RET negative. For CAT and metabolites in plasma, upper reference limits are (in nmol/l); NE (norepinephrine): 6.55, E (epinephrine): 1.23, free NMN (normetanephrine): 1.39, free MN (metanephrine): 0.85, P65 was later shown not to be a PHEO and do not appear in this table

### Quantitative PCR, western blot and antibodies

PHEO/PGL samples were prepared devoid of remaining healthy tissue by the surgeon or the pathologist. RNA extraction was performed using Trizol (Invitrogen, Luzern, Switzerland), and cDNA synthesis using the PrimeScript RT reagent Kit (Takara Bio Inc, Japan). Primers were chosen with the primer designing tool from the National Center for Biotechnology Information (NCBI) and described elsewhere [13]. Real time PCR assays were conducted in 384 wells using Sybergreen (Roche, Basel, Switzerland) with an Applied Biosystems 7900HT SDS (Life technology, Zug, Switzerland). Negative controls were performed on the same amount of total RNA without adding the reverse transcriptase (NEC, non enzyme control). All assays were tested on a cDNA dilution serie with efficiency comprised between 0.98 and 1.0. qPCR parameters were: 95°C 10 min, 40 cycles 95°C 15 sec. 60°C 1 min and melting curves were as expected in all cases. Normalisation of gene expression was performed on three reference genes; TBP (tata-box binding protein), Eef1a1 (eukaryotic translation elongation factor 1 alpha 1) and GAPDH (glyceraldehyde-3-phosphate dehydrogenase). Amplification curves and fold changes were analysed using the REST 2009 software (Qiagen, Hombrechtikon, Switzerland). For western blot assays, tissues were disrupted using a micro potter in a lysis buffer containing PBS with 0.5% triton X-100 and protease inhibitor with EDTA (Complete, Roche) to represent 20% w/v. Samples were fractionated by SDS-PAGE under reducing conditions using 12% precast gels (Bio-Rad, Reinach, Switzerland). Proteins were electroblotted onto nitrocellulose membrane and probed with antibodies. Immunoreactive bands were revealed by chemiluminescence assay (PerkinElmer, Schwerzenbach, Switzerland) and the signal was processed by a digital imaging analyzer (ImageQuant LAS-4000, General Electric, Glattbrugg, Switzerland) and quantified with the corresponding software (multigauge, General Electric). Quantification values were given as QL (pixel value)—background (BG). Anti-PNMT polyclonal antibody raised in rabbit (final dilution 1000x) was purchased from Protos (cat. number CA-401, Protos Biotech Corp. New York, USA), anti-β-actin mouse monoclonal antibody (final dilution 5000x) was from Sigma (AC-15, cat. number A5441, Sigma-Aldrich-Chemie, Buchs, Switzerland) and anti-TH polyconal antibody raised in rabbit (final dilution 1000x) from Millipore (cat. number AB152, Millipore, Zug, Switzerland). Anti-DBH polyconal antibody raised in rabbit (final dilution 5000x) was generated against full length recombinant human DBH protein [14]. Secondary HRP conjugated anti-mouse and anti-rabbit antibodies were from Bio-Rad (cat. number 170–6516 and 170–6515, respectively and diluted 4000x).

### Primary cell culture

Tumors were cut in small pieces and digested with collagenase (1mg/ml) (Sigma) in Dulbecco's modified Eagle medium (DMEM, Life technology), under agitation at 37°C until complete dissolution of tumor pieces. Cells were washed by centrifugation (235g for 2 minutes), suspended in DMEM supplemented with 10% fetal bovine serum (Life technology) and platted in 24 well plates with 1μM dexamethasone (Sigma) in DMEM. After 24 hrs incubation in a humidified, 5% CO_2_ incubator at 37°C, cells were lysed in 200μl lysis buffer.

### CAT and MNs extraction and quantification in tissue and plasma

Tumor tissues were disrupted in perchloric acid 0.1M and were sonicated using a Branson Sonifier 450 (Branson, Danbury, CT, USA) at full power for 30 seconds. CAT (E and NE), were extracted using activated alumina in 0.5 ml microcentrifuge tubes and quantified by ultraperformance liquid chromatography-tandem mass spectrometry (UPLC-MS/MS) [15]. Metanephrines (MN and NMN) were extracted and quantified as previously published [16]. Values are expressed in nanomoles of metabolite per gram of tissue or per liter of plasma.

### Statistics

Data was studied using Graph Pad prism 6.0. The differences in CAT, MNs and protein concentration (in tissue and/or in plasma) between different types of tumors were assessed using the t-test unpaired and Mann-Whitney nonparametric test. Differences were considered as significant when *P*<0.05. mRNA expression was quantified and statistically explored with the REST2009 software from Qiagen.

## Results

### CAT levels in tumor and plasma of PHEO and PGL affected patients

**Fig 2:**
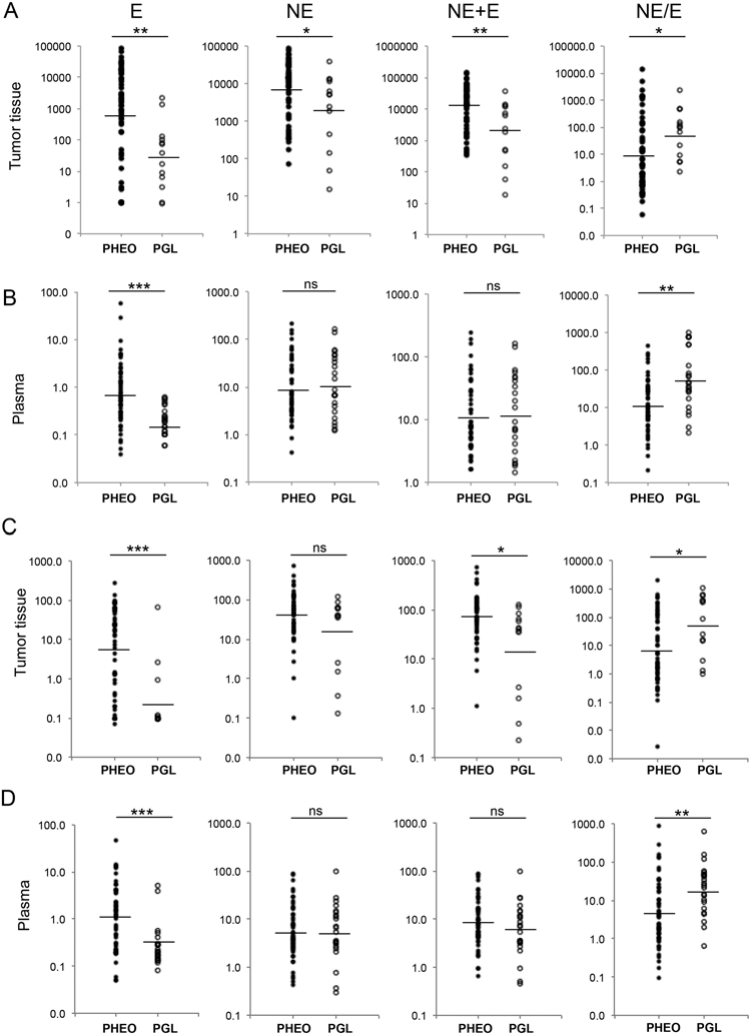
Quantification of E, NE, MN and NMN in plasma and tumor tissue. (A) Quantification of epinephrine (E), norepinephrine (NE), total CATs (E+NE) and NE/E ratio from 53 PHEO (filled circles) and 13 PGL (open circles) samples. Results are given in nanomoles per gram of tissue and represented on a logarithmic scale. Geographic mean and standard deviation for each group are reported in supporting information (S2 Table). (B) Same as in A, quantification of CAT in plasma for 50 PHEO and 23 PGL. (C) Quantification of metanephrine (MN), normetanephrine (NMN), sum of metanephrines (MN+NMN) and NMN/MN ratio from 53 PHEO (filled circles) and 13 PGL (open circles).(D) Same as in C, quantification of metanephrines in plasma for 51 PHEO and 23 PGL. ***P<0.001, **P<0.01, *P<0.05, ns: not statistically significant. For CAT and metanephrines in tumor tissue, minimal values were set at 0.1 nmol/g representing limit of quantification.

**Fig 3:**
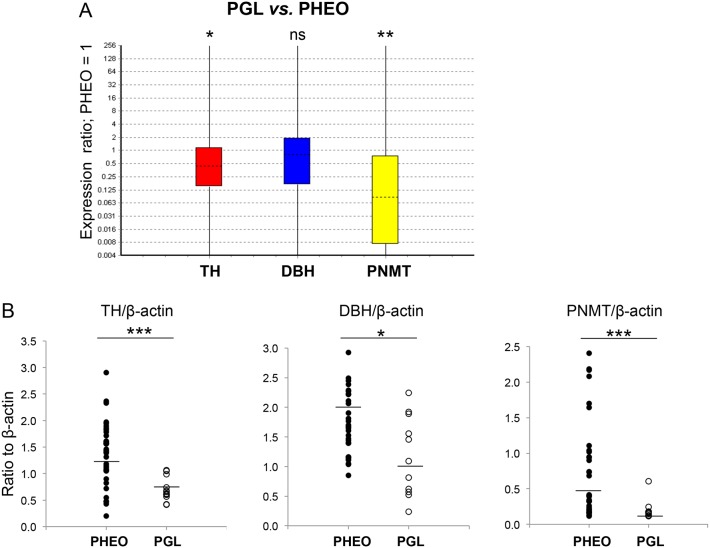
mRNA and protein quantification of TH, DBH and PNMT in PGL *vs*. PHEO. (A) Whisker-box plot of mRNA relative ratio from 11 PGL *vs*. 50 PHEO (reference). TH: fold change to reference (FC) 0.38, Standard error (SE) 0.08–1.94, 95% confidence Interval (CI) 0.003–13.37; PNMT: FC 0.095, SE 0.003–2.35, CI 0–74.06, DBH: not statistically significant (ns). Reporter genes included the following: EEFIA1, GAPDH and TBP. ***P<0.001, **P<0.01, *P<0.05. (B) Quantification of the signal obtained by western blot for 35 PHEO (filled circles) and 12 PGL (open circles). Results are given as QL (corresponding band pixel values)-BG (background pixel value), and calculated values were divided by the β-actin signal value and reported on the y-axis. Geographic mean and standard deviation for each group are reported in supporting information (S3 Table). ***P<0.001, **P<0.01, *P<0.05, ns; not statistically significant.

We observed a lower intra-tumoral concentration of both E and NE per gram of tissue in PGL compared with PHEO (by 25.6 and 4.2 fold respectively, *P*<0.01 and *P* = 0.02) (Fig 2A). Lower CAT concentration in PGL was associated with TH and PNMT mRNA lower abundance; TH was downregulated by 2.6 fold *P* = 0.04 and PNMT by 10.5 fold *P*<0.01 compared with PHEO, (Fig 3A) mRNA data were confirmed at the protein level with 1.8 fold, *P*<0.001 and 2.8 fold, *P*<0.001, expression decrease, respectively (Fig 3B). However due to the high heterogeneity of tumors for CAT content and secretion (Fig 2A and 2B), PHEO were further subdivided into two groups; PHEO were considered as noradrenergic (NorAd PHEO) when the E concentration in tumor tissue was below 10% of total CAT (NE+E), and tumors with E concentration above 10% of total CAT were termed as mixed PHEO (Table 1) as previously published [12]. Hence by comparing these three groups, if differences between PHEO and PGL exist, then PGL group would clearly be different from both PHEO groups (noradrenergic and mixed) regarding CAT metabolism.

**Fig 4:**
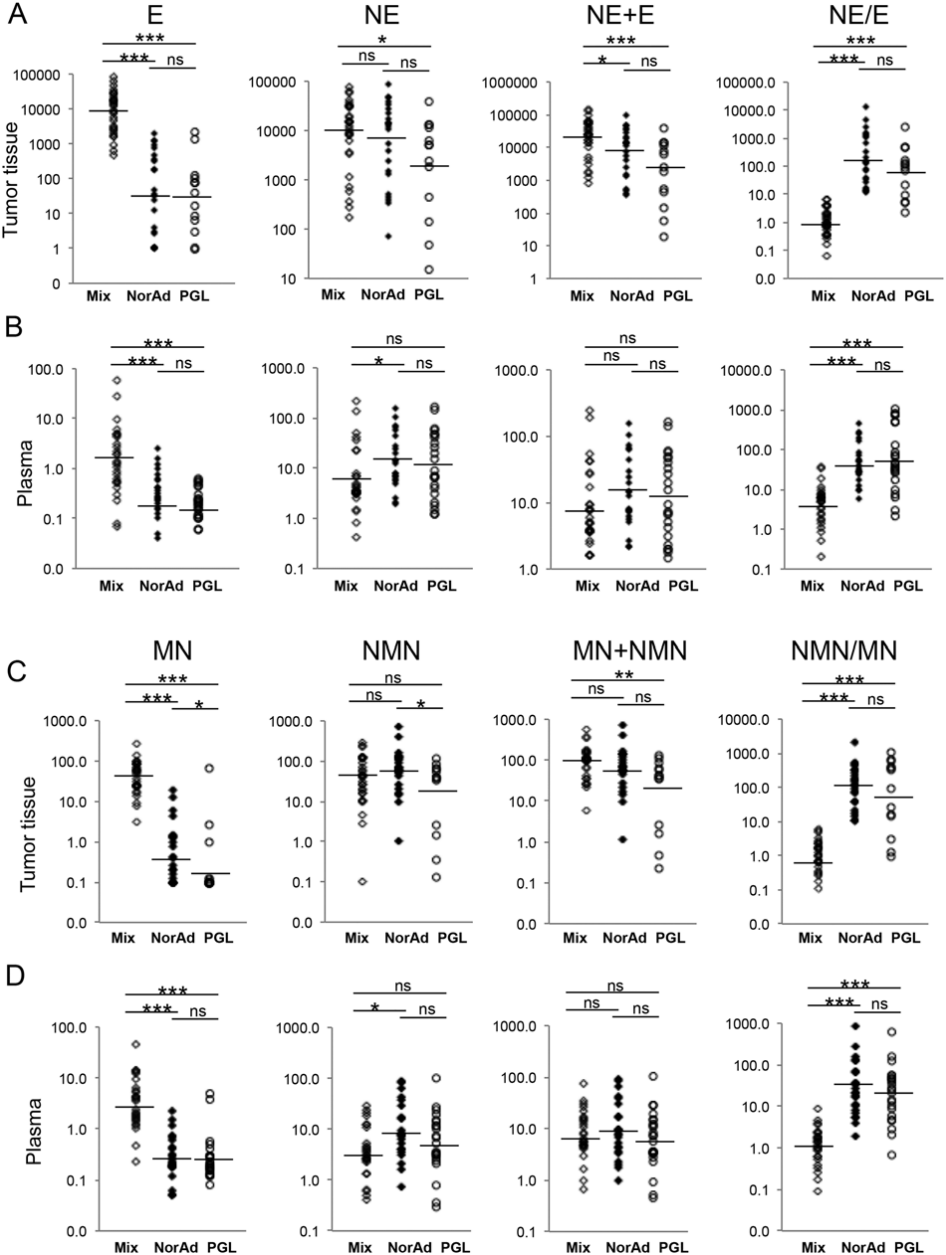
Quantification of E, NE, MN and NMN in plasma and tumor tissue of PGL, mixed and NorAd tumors. (A) Quantification of epinephrine (E), norepinephrine (NE), total CAT (E+NE) and NE/E ratio from 30 mixed PHEO (Mix, open diamonds); 23 noradrenergic PHEO (NorAd, filled diamonds) and 13 PGL (open circles) samples. Results are given in nanomoles per gram of tissue and represented on a logarithmic scale. Geographic mean and standard deviation for each group are reported in supporting information (S4 Table). (B) Same as in A, quantification of CAT in plasma for 28 mixed PHEO, 22 NorAd PHEO and 23 PGL. (C) Quantification of MN and NMN sum of MNs (MN+NMN) and NMN/MN ratio from 30 mixed PHEO (Mix, open diamonds); 23 NorAd PHEO (filled diamonds) and 13 PGL (open circles) samples. (D) Same as in C, quantification of MNs in plasma for 28 mixed PHEO, 22 NorAd PHEO and 23 PGL. ***P<0.001, **P<0.01, *P<0.05, ns: not statistically significant. For CAT and MNs in tumor tissue, minimal values were set at 0.1 nmol/g representing limit of quantification.

E concentration and NE/E ratio in tumor tissue were similar between NorAd PHEO (n = 23) and PGL (n = 13), while PGL had 261 fold less E concentration compared with mixed PHEO (n = 30) *P*<0.0001 (Fig 4A). Total CAT concentration was 9.5 fold lower in PGL compared with mixed tumor *P*<0.001 but not statistically different from NorAd PHEO (Fig 4A). A similar correlation was recorded in plasma for E concentration (7 fold decreased in PGL compared to mixed PHEO *P*<0.001, Fig 4B). No significant difference were observed between PHEO and PGL (Figs 2B and 4B) for NE and sum of CAT (NE+E), due to the fact that neuronal NE production accounts for most of NE circulating levels while most of plasma E arises from adrenal synthesis.

### Metanephrine concentration in tumor and plasma of PHEO and PGL affected patients

We then investigated the possible differences regarding CAT metabolism with respective MN and NMN content in PHEO and PGL for tumor and plasma concentration. In tumor tissue as well as in plasma, MN content was lower in PGL (by respectively 20, *P*<0.0001 and 3.3 fold *P* = 0.0001) than in PHEO (mixed and NorAd tumors) (Fig 2C and 2D). NMN concentration was not different between PGL and PHEO due to neuronal NE production and recaptation by pheochromocytes. When PHEO were partitioned into mixed and NorAd tumors, there was a much higher tissue concentration of MN in mixed PHEO compared to PGL (148 fold increase, *P*<0.0001) and a slight difference when PGL group was compared with NorAd PHEO (approx. 2 fold increase, *P* = 0.04) (Fig 4C). A significant difference was also observed between these two types of tumors (PGL and NorAd) for NMN concentration (4 fold difference *P* = 0.04), while the sum of metanephrines (MN+NMN) was not different between NorAd PHEO and PGL and slightly less abundant in PGL compared to mixed PHEO (3.6 fold decrease, *P* = 0.01) (Fig 4C). In plasma, metanephrine concentration patterns were similar to those monitored for CAT plasma concentration (Fig 4D compared with Fig 4B).

From a genetic point of view, among the 13 patients affected by a PGL from our cohort, 4 tumors were the consequence of an SDHB mutation and 5 were not SDHB or VHL related. NE and E tissue concentration were not statistically different between patients affected by a PGL with an SDHB mutation and patients with a PGL not arising from an SDHB or VHL mutation. (S5 Table).

### mRNA and protein quantification of TH, DBH and PNMT

**Fig 5:**
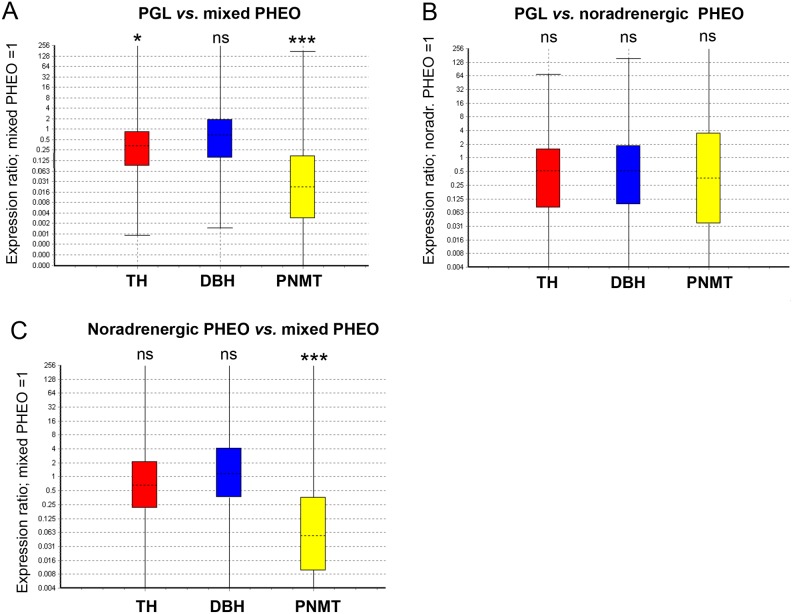
mRNA and protein quantification of TH, DBH and PNMT in PGL *vs*. mixed and NorAD PHEO. (A) Whisker-box plot of mRNA relative ratio from PGL (n = 11) *vs*. mixed PHEO (n = 28) (reference). TH: reaction efficiency (RE) 0.99, fold change to reference (FC) 0.29, Standard error (SE) 0.04–1.65, 95% confidence Interval (CI) 0.003–48.29; DBH: not statistically significant (ns); PNMT: RE 0.98, FC 0.024, SE 0.001–0.41, CI 0–4.82. Reporter genes included the following: EEFIA1, RE 0.99; GAPDH, RE 1.04, TBP, RE 0.97. ***P<0.001, **P<0.01, *P<0.05, (B) Ratio from PGL (n = 11) *vs*. NorAd PHEO (n = 22) (reference). (C) Ratio from NorAd PHEO (n = 22) *vs*. mixed PHEO (n = 28) (reference) PNMT: FC 0.07, SE 0.005–0.93, CI 0.001–10.99.

In order to explain differences in CAT concentration and composition within the three groups of tumors, gene expression of *TH*, *DBH* and *PNMT* was assessed by qPCR. *TH* and *PNMT* genes were down regulated in PGL compared with mixed PHEO by 3.5 and 41.7 fold respectively (*P* = 0.03 and *P* = <0.001) while no differences were recorded for *DBH* (Fig 5A) As observed for E and NE concentration in tumor tissue, there was no significant difference between CAT synthesis genes expression for PGL and NorAd PHEO (Fig 5B). *PNMT* was also highly down regulated in NorAd PHEO compared to mixed PHEO (Fig 5C), in accordance with low E measured compared with mixed tumor (15.4 fold, *P*<0.001). In contrast with *TH* expression in PGL *vs*. mixed PHEO, *TH* in NorAd PHEO was not shown to be down-regulated when compared with mixed PHEO (Fig 5A compared with Fig 5C), even though NorAd tumors contain significantly less total CAT than mixed tumors.

**Fig 6:**
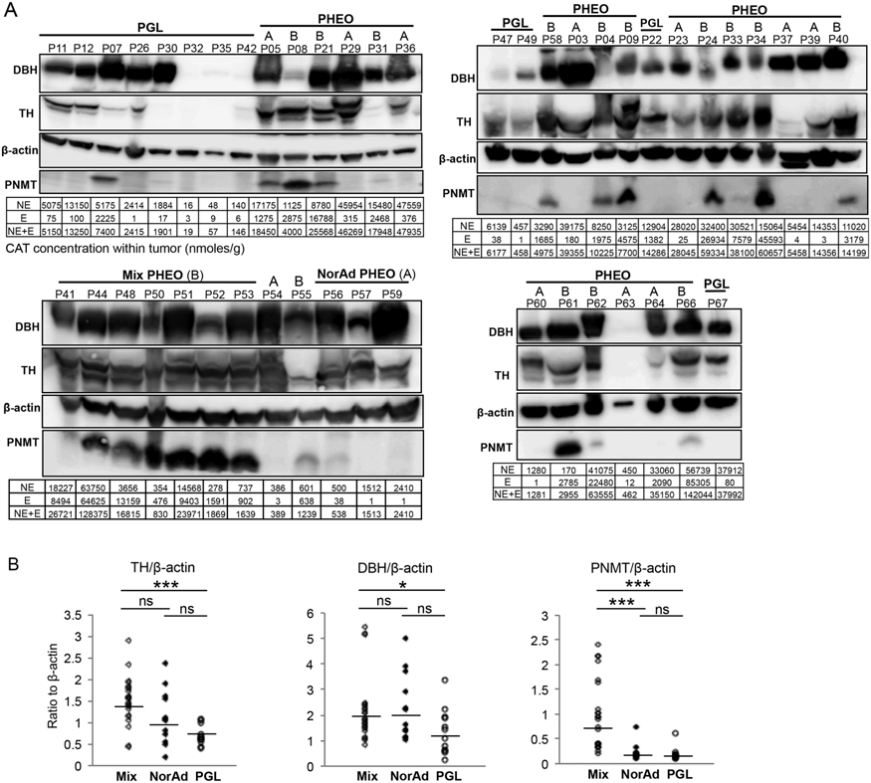
TH, DBH and PNMT protein expression in tumors. (A) Western blot of sample tissues from NorAd PHEO (A, n = 14), mixed PHEO (B, n = 21) and PGL (n = 12). Volumes loaded were 10μl of a 20% w/v extract. E and NE values corresponding to the samples analysed by western blot are given in nanomoles/gram of tissue, lower quantification limit was set at 1nmole/gram. (B) Quantification of the signal obtained by western blot. Results are given as QL (corresponding band pixel values)-BG (background pixel value), and calculated values are divided by the β-actin signal value and reported on the y-axis. Geographic mean and standard deviation for each group are reported in supporting information (S6 Table). ***P<0.001, **P<0.01, *P<0.05, ns; not statistically significant.

To ensure that mRNA and protein concentration were correlated, TH, DBH and PNMT protein expression was measured by western blot among the tumor samples of the three groups. Signal obtained was normalised with β-actin band intensity (Fig 6A). TH and PNMT protein expression reflected mRNA tumor concentration; both proteins were shown to be less abundant in PGL compared to mixed PHEO by respectively 2.1 and 4.4 fold (*P*<0.001 for both correlation) (Fig 6B). DBH was slightly down-regulated in PGL compared to mixed PHEO by 1.8 fold, *P* = 0.03 contrasting with mRNA concentration that was unchanged (Fig 5). Interestingly, no significant changes were observed at both mRNA and protein level for the three genes studied between PGL and NorAd PHEO (Figs 5B and 6B). PNMT protein expression was only detected in one PGL (P07) in accordance with relatively high E tissue levels for this sample (Fig 6A). CAT values for each tumor were reported under respectively TH, DBH and PNMT expression signal which showed that in most of tumors, low E level corresponds to undetectable PNMT signal.

### PNMT expression and E synthesis: Correlation with mixed and NorAd PHEO

**Fig 7:**
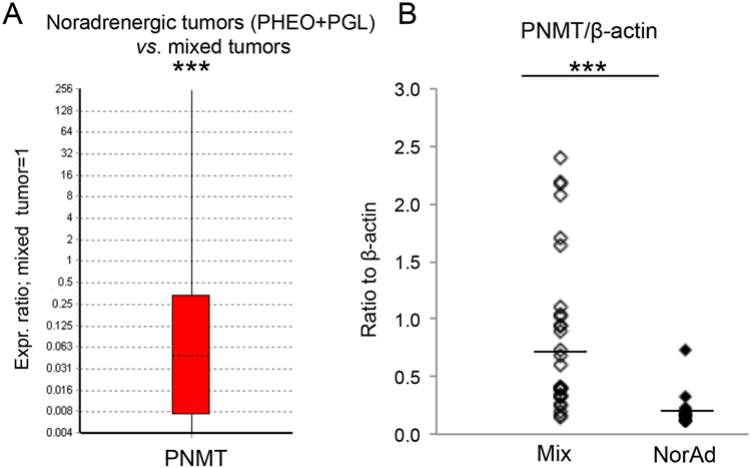
PNMT expression in NorAd PHEO *vs*. mixed tumors. (A) Whisker-box plot of PNMT mRNA relative ratio from NorAd tumors (including PHEO and PGL, n = 29) *vs*. mixed tumor (PHEO and PGL, n = 32, reference). Fold change to reference (FC) 0.06, Standard error (SE) 0.003–0.29, 95% confidence Interval (CI) 0–22.36; Reporter genes included the following: EEFIA1, GAPDH and TBP. (B) Quantification of the signal obtained by western blot in Fig 6 for PNMT signal in mixed and NorAd tumor (independently of PHEO or PGL). Results are given as QL (corresponding band pixel values)-BG (background pixel value) and calculated value is divided by the β-actin signal value and reported on the y-axis. Geo. mean 0.64 *vs*. 0.18, standard deviation: 0.7 and 0.14 for respectively mixed and NorAd tumor.

Hitherto no report has directly shown the correlation between PNMT protein expression and E concentration in PHEO/PGL tissue, and it remained to be demonstrated in our study that low PNMT is directly correlated with low E synthesis independently of tumor localisation. Hence, PNMT expression was assessed at both mRNA and protein levels by qPCR and WB in the same set of PHEO analysed for protein expression from Fig 6. Results showed an increase of 59 fold, (*P*<0.001) for mRNA expression in mixed *vs*. NorAd tumor (Fig 7A) and protein expression was 3.6 fold up-regulated in mixed PHEO (Fig 7B). This indeed demonstrates that, independently of tumor localisation, low PNMT levels in PGL and PHEO, at both mRNA and protein levels, account for low E concentration.

### Effects of glucocorticoids on TH, DBH and PNMT gene transcription and protein expression

**Fig 8:**
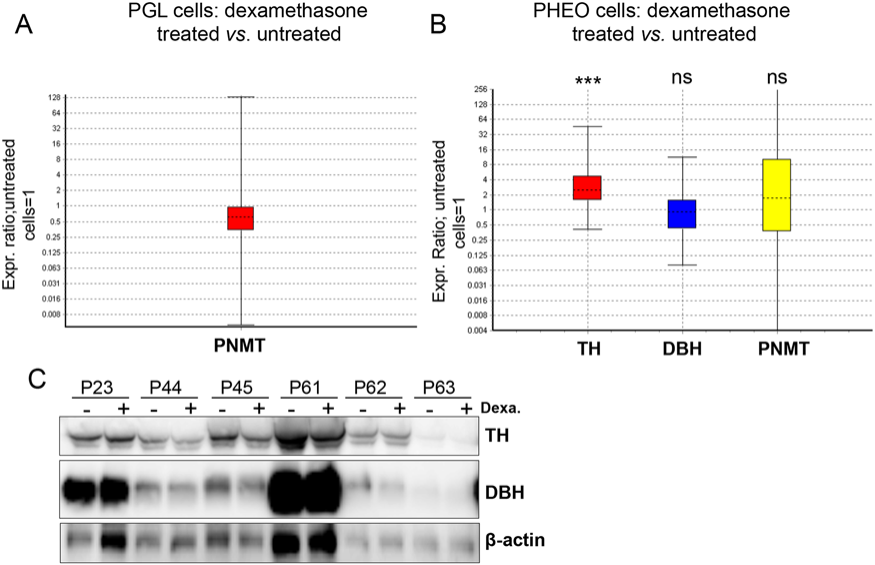
Dexamethasone effects on primary tumor cells. (A) Whisker-box plot of PNMT mRNA relative ratio from PGL cells (tumors P16, P30, P35 and P42) incubated with *vs*. without dexamethasone 1μM for 24 hrs (reference). Reporter genes included the following: EEFIA1, GAPDH and TBP, ns; not statistically different. (B) Whisker-box plot of TH, DBH and PNMT mRNA relative ratio from PHEO cells (tumors P23, P36, P44, P45, P52, P53, P56, P61 and P62) incubated with *vs*. without dexamethasone 1μM for 24 hrs (reference). TH: FC 2.8, SE 1.28–7.55, CI 0.68–17.38, reporter genes included the following: EEFIA1, GAPDH and TBP. ** *P*<0.01. (C) Western blot of PHEO cell incubated with (+) or without (-) dexamethasone (Dexa.) 1μM for 24 hrs.

To assess experimentally the effects of GC on *PNMT*, *TH* and *DBH* gene expression in primary cell culture, cells from 4 PGL and 9 PHEO (6 mixed and 3 NorAd tumors) were plated in culture dishes and incubated with or without dexamethasone for 24 hrs. In PGL cells no significant effect on PNMT mRNA was detected. DBH and TH mRNA were below quantification limit due to low material amount available after surgical resection and lower qPCR efficiency for these two genes compared to PNMT (Fig 8A). In PHEO cells dexamethasone induced a 2.8 fold upregulation of *TH* compared with non dexamethasone-incubated cells at 24 hrs (P = 0.002), no effect was recorded for *DBH* and *PNMT* (Fig 8B) and between mixed (n = 6) *vs*. noradrenergic PHEO (n = 3) regarding gene transcription for *TH*, *DBH and PNMT* after 24 hrs incubation with dexamethasone. To correlate gene with protein expression, TH, DBH and PNMT protein concentration was revealed from dexamethasone incubated and control cells after 24hrs incubation. Absence of activation was confirmed for *DBH* and *PNMT* in steroids-incubated cell compared to control cells while *TH* up-regulation detected for mRNA was not confirmed at the protein level.

## Discussion

By comparing PHEO and PGL CAT content, genes expression and protein concentration we aimed to differentiate CAT metabolism in tumor cells developing in a GC-rich environment with tumors that lost anatomical contact with adrenal cortex.

We first observed that PGL produce and secrete lower amounts of CAT and MNs, especially E and MN than PHEO tumors occurring in the adrenal gland. It is commonly supposed that low epinephrine and PNMT concentrations may arise from a disruption of cellular communication between pheochromocytes from PGL and adrenal cortex. Low GC level may not be able to transactivate a large array of genes that possess GC-binding sites including PNMT [6–12] and TH [17]. However, when PHEO were divided into two groups, according to tissue CAT NE/E ratio, a different scenario took shape; PGL and NorAd PHEO featured similar values without significant differences regarding all aspects studied (tissue and plasma CAT concentration as well as gene and protein expression involved in CAT synthesis). This demonstrates that tumor characteristics, at least regarding CAT production, are not driven by the immediate environment where the tumor develops, minimising the need of high GC gradients coming from cortical cells. Furthermore, tumor size was not correlated with NorAd, mixed PHEO or PGL (S7 Table).

Our results suggest that the initial tumor cell generated presents characteristics that will not change depending on the environment and location of incidence. Steroids or other factors from adjacent cortical cells may however have an effect on CAT gene activation, since 54% of PHEO (19/35) show PNMT activation, contrasting with only one PGL tumor (1/12, 8%) where PNMT is detected (Fig 6A). PNMT expression in PGL has also been reported elsewhere ranging from 1/12 cases [18], similar with our results, to 9/12 cases [19]. We show in this report that direct contact with cortical cells is in no case sufficient to transactivate target genes since NorAd PHEO and PGL sustain similar *TH*, *DBH* and *PNMT* activation. Both kinds of tumors may have common transcriptional dysregulation independently of steroid or cortical factors activation. In this scenario oncogenesis occurs before differentiation into chromaffin cells as illustrated by *PNMT* gene regulation; NorAd PHEO and PGL produce similar amounts of mRNA while NorAd and mixed PHEO, both in contact with the cortex, show a drastic difference in PNMT mRNA concentration (Fig 5C). *In vivo PNMT* activation requires the addition of several factors, as recently reviewed by Kvetnansky and colleagues [20]. However, GC receptors and transcription factors including Early growth response protein 1 (Egr-1), Specificity protein 1 (Sp1) and Myc-associated zinc finger protein (MAZ) known to activate *PNMT* were shown not to be differentially expressed between adrenergic (Men2a) versus non adrenergic (VHL) tumors [21]. Hypermethylation of *PNMT* has been recently demonstrated in SDH and VHL-related tumors that led to a downregulation of mRNA synthesis [22, 23]. We confirmed at the protein level the epigenetic silencing of *PNMT* (all tested tissue samples from SDHB- and VHL- associated tumors had undetectable PNMT level, Table 1 and Fig 6A), suggesting that hypermethylation may also explain lack of PNMT protein expression in noradrenergic PHEO independently of identified mutation

Our results demonstrate that neither *in vivo* (direct contact between pheochromocytes and cortical cells) nor *in vitro* (pheochromocytes treated with dexamethasone) is sufficient to ensure *PNMT* transcription. This contrasts with chromaffin cells from mice and bovines where treatment with 100nM to 20 μM of dexamethasone results in high *PNMT* transactivation levels [24, 25]. We report here an upregulation of *TH* transcription level following dexamethasone incubation without effect on final protein concentration.

Pheochromocytoma crisis (PC) induced by GC is a major concern in PHEO-suspected patients: several PC cases have been reported following corticosteroid administration [26–28], on the other hand, GC administration in patient without PHEO was not correlated with changes in plasma CAT and MNs [29]. We demonstrate in the present study that primary PHEO cells in culture incubated with 1μM of dexamethasone during 24 hrs did not result in a higher production of the main enzymes involved in CAT metabolism, suggesting that GC has no direct impact on long term regulation of catecholamine synthesis in PHEO. The lag phase (5–36 h) recorded in the majority of reported cases of PC argues against a direct effect on CAT release as discussed by Druce and colleagues [29] and could have suggested a genetic regulation by exogenous GC, but we show in this report that it is unlikely. PC is therefore probably due to other factors (drugs therapy and interaction, trauma surgery) rather than GC treatment [29].

Another aspect of our work was to potentially differentiate between PHEO and PGL by comparing CAT and MN plasma levels of affected patients. This issue is of great interest for endocrinologists receiving positive results from blood tests, providing an initial idea about possible tumor location before imaging examination. In a previous report Eisenhofer and colleagues showed that a high concentration of plasma free metanephrine relative to normetanephrine indicates an adrenal location [30]. We also observed a similar correlation (Fig 2D, right panel) but when PHEO were partitioned as mixed and noradrenergic tumor and compared to PGL, no differences were detected between noradrenergic PHEO and PGL (Fig 4D right panel). Hence, no correlations were found that could biochemically differentiate PGL from noradrenergic PHEO, giving another piece of evidence that tumor environment may only play a minor role in PHEO/PGL CAT metabolism.

In conclusion, a specific biochemical phenotype is not determined by the tumor location and the postulated effect of a strong GC gradient on CAT synthesis and more specifically on E production is not supported by our work.

## Supporting Information

S1 Table Mutations identified in PHEO/PGL patients from Table 1. Neg: no mutation identified for the gene considered, NT: gene not screened. *: family and/or clinical history of mutation for the gene considered, no additional tests conducted. **: mutation on VHL gene identified in Paris in 1999.
(TIF)

S2 Table For Fig 2. Quantification of catecholamine and metanephrines in PHEO and PGL tissue and plasma. Geographic mean and standard deviation for the values reported.
(TIF)

S3 Table For Fig 3. Quantification of TH, DBH and PNMT expression in PHEO and PGL tissue and plasma. Geographic mean and standard deviation for the values reported.
(TIF)

S4 Table For Fig 4. Quantification of catecholamine and metanephrines in mix-, NorAd- PHEO and PGL in tissue and plasma. Geographic mean and standard deviation for the values reported.
(TIF)

S5 Table Plasma and tissue concentration of CAT and MNs from PGL unrelated to SDHB/VHL mutation and SDHB PGL. (TIF)

S6 Table For Fig 6B. Quantification of TH, DBH and PNMT expression in mix-, NorAd- PHEO and PGL tissue. Geographic mean and standard deviation for the values reported.
(TIF)

S7 Table Tumor size of mixed PHEO, NorAd PHEO and PGL. Values were taken from Table 1. When more than one dimension was available, mean value was calculated and reported. No significant differences were observed between the three groups.
(TIF)
