# Supplementary figures and images for: Catecholamine Metabolism in Paraganglioma and Pheochromocytoma: Similar Tumors in Different Sites?
Source: PLoS One. 2015 May 6;10(5):e0125426. doi: 10.1371/journal.pone.0125426 (PMC4422698; doi:10.1371/journal.pone.0125426)

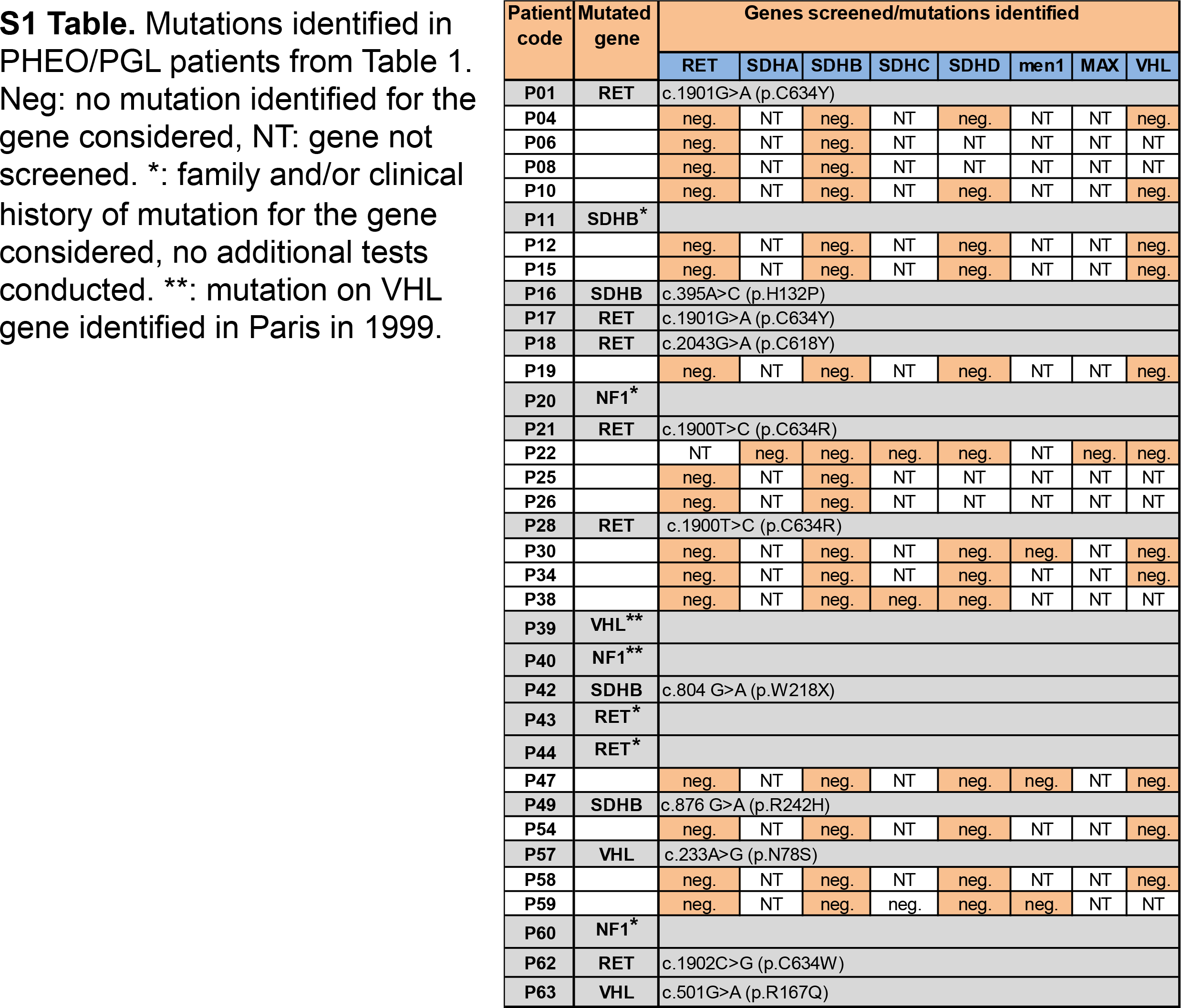

Supplement: S1 Table — Neg: no mutation identified for the gene considered, NT: gene not screened. *: family and/or clinical history of mutation for the gene considered, no additional tests conducted. **: mutation on VHL gene identified in Paris in 1999. (TIF) [file pone.0125426.s001.tif]

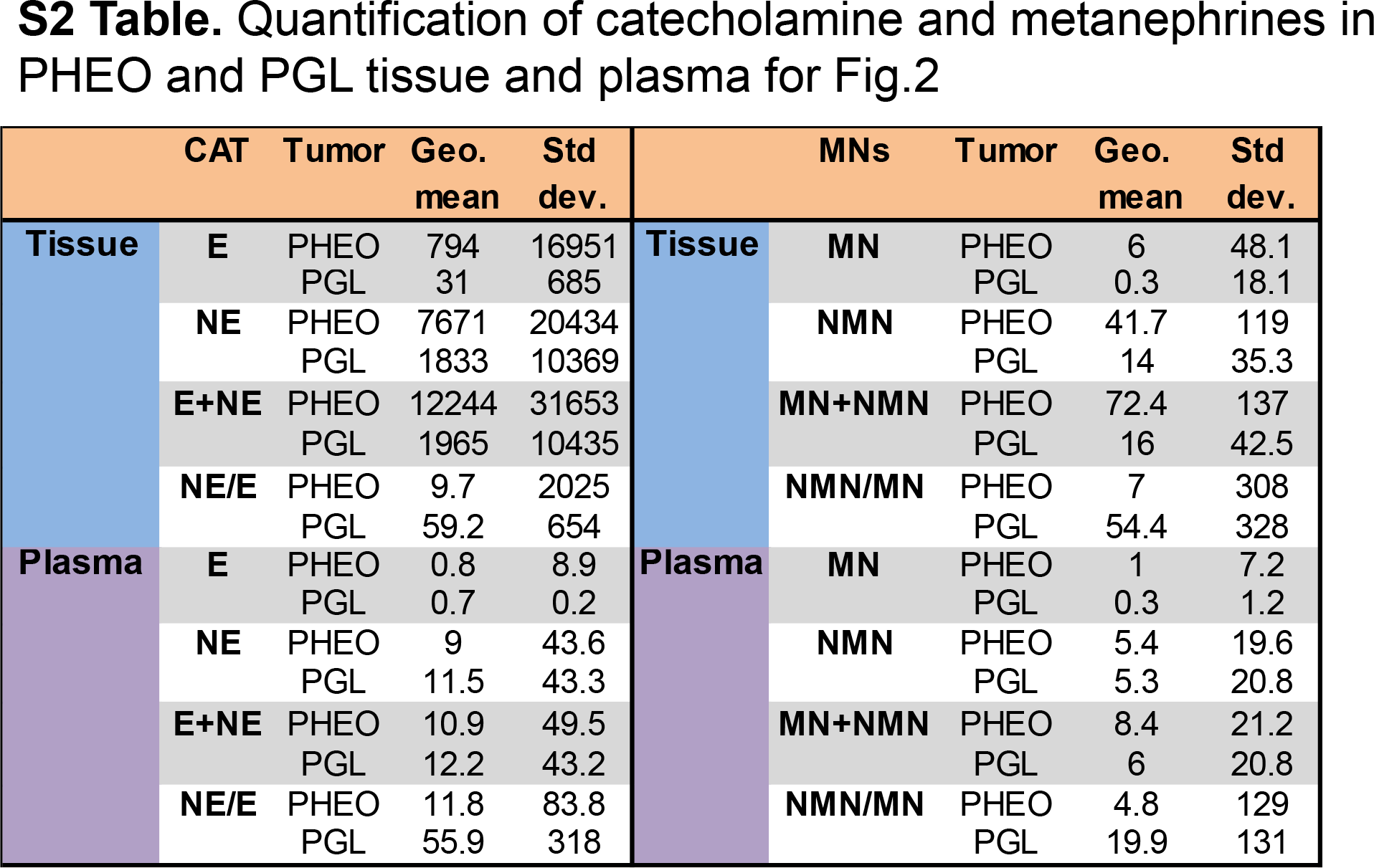

Supplement: S2 Table — Quantification of catecholamine and metanephrines in PHEO and PGL tissue and plasma. Geographic mean and standard deviation for the values reported. (TIF) [file pone.0125426.s002.tif]

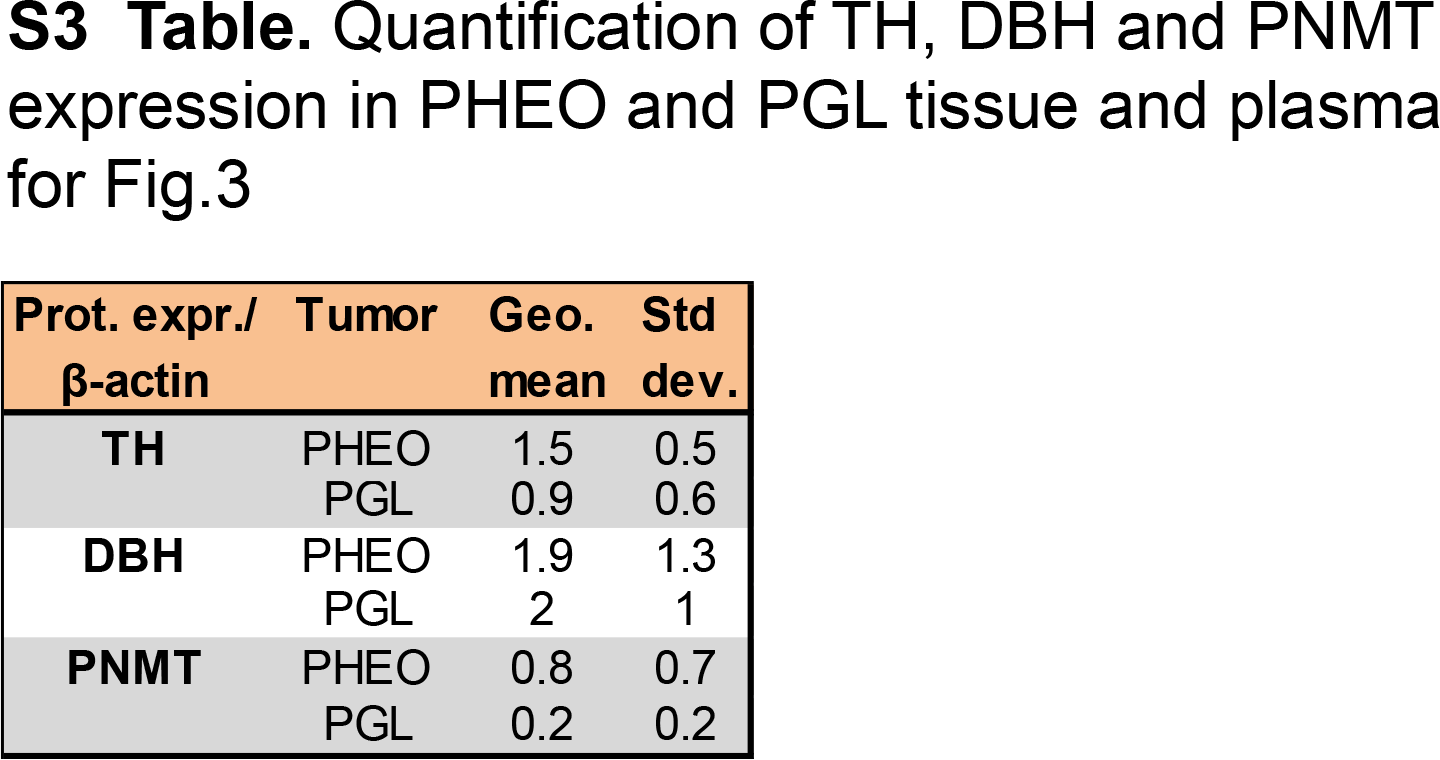

Supplement: S3 Table — Quantification of TH, DBH and PNMT expression in PHEO and PGL tissue and plasma. Geographic mean and standard deviation for the values reported. (TIF) [file pone.0125426.s003.tif]

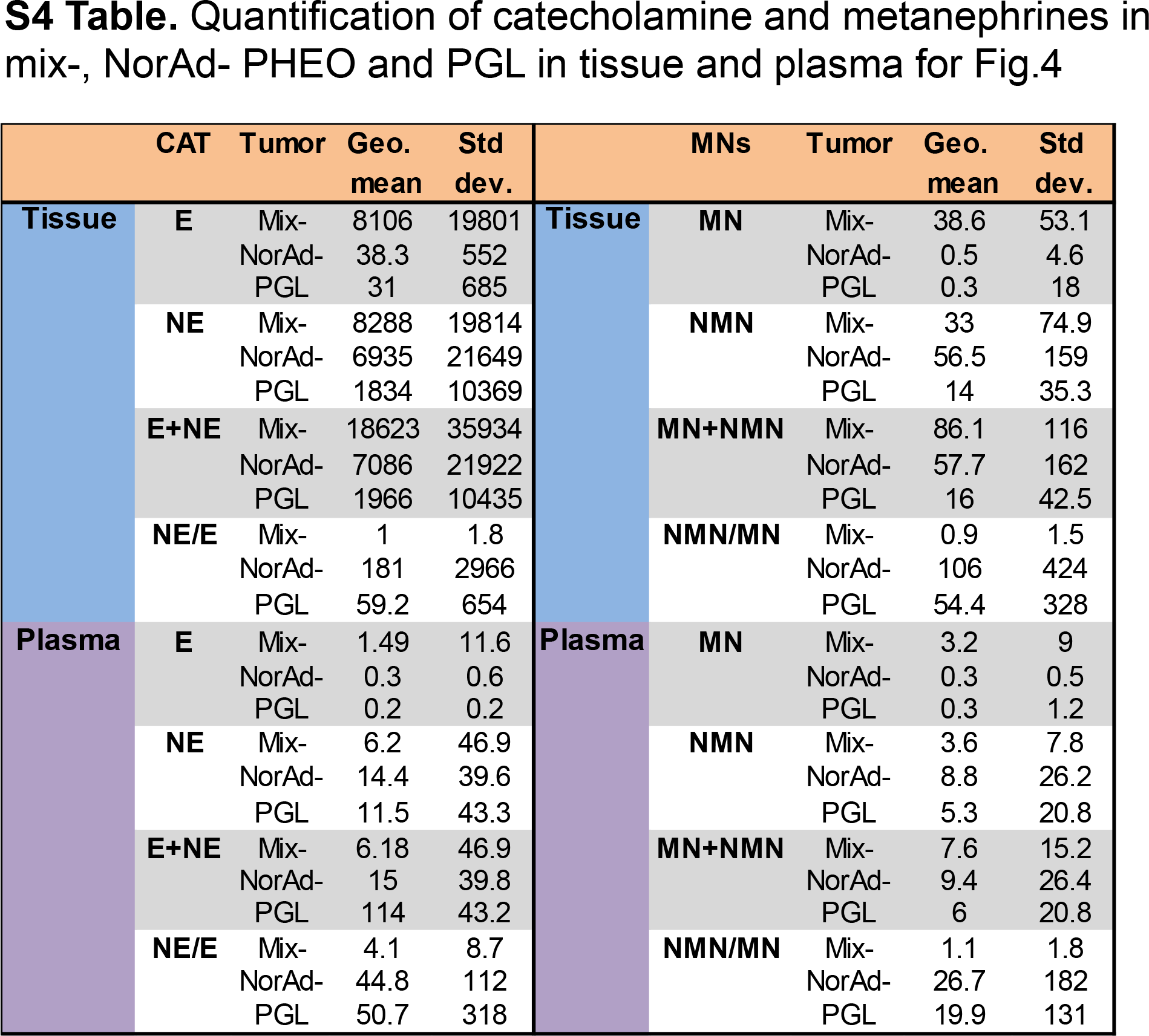

Supplement: S4 Table — Quantification of catecholamine and metanephrines in mix-, NorAd- PHEO and PGL in tissue and plasma. Geographic mean and standard deviation for the values reported. (TIF) [file pone.0125426.s004.tif]

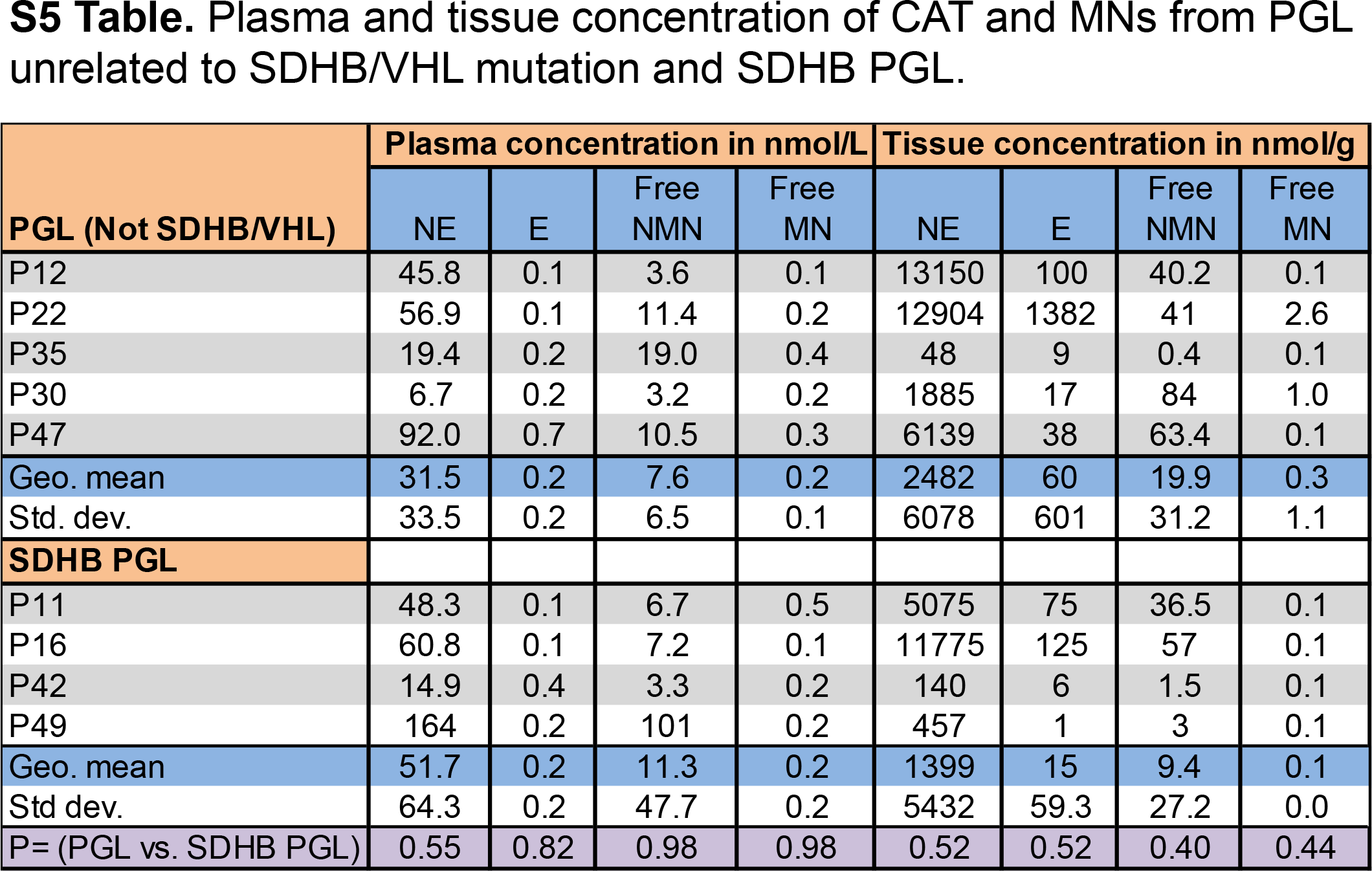

Supplement: S5 Table — (TIF) [file pone.0125426.s005.tif]

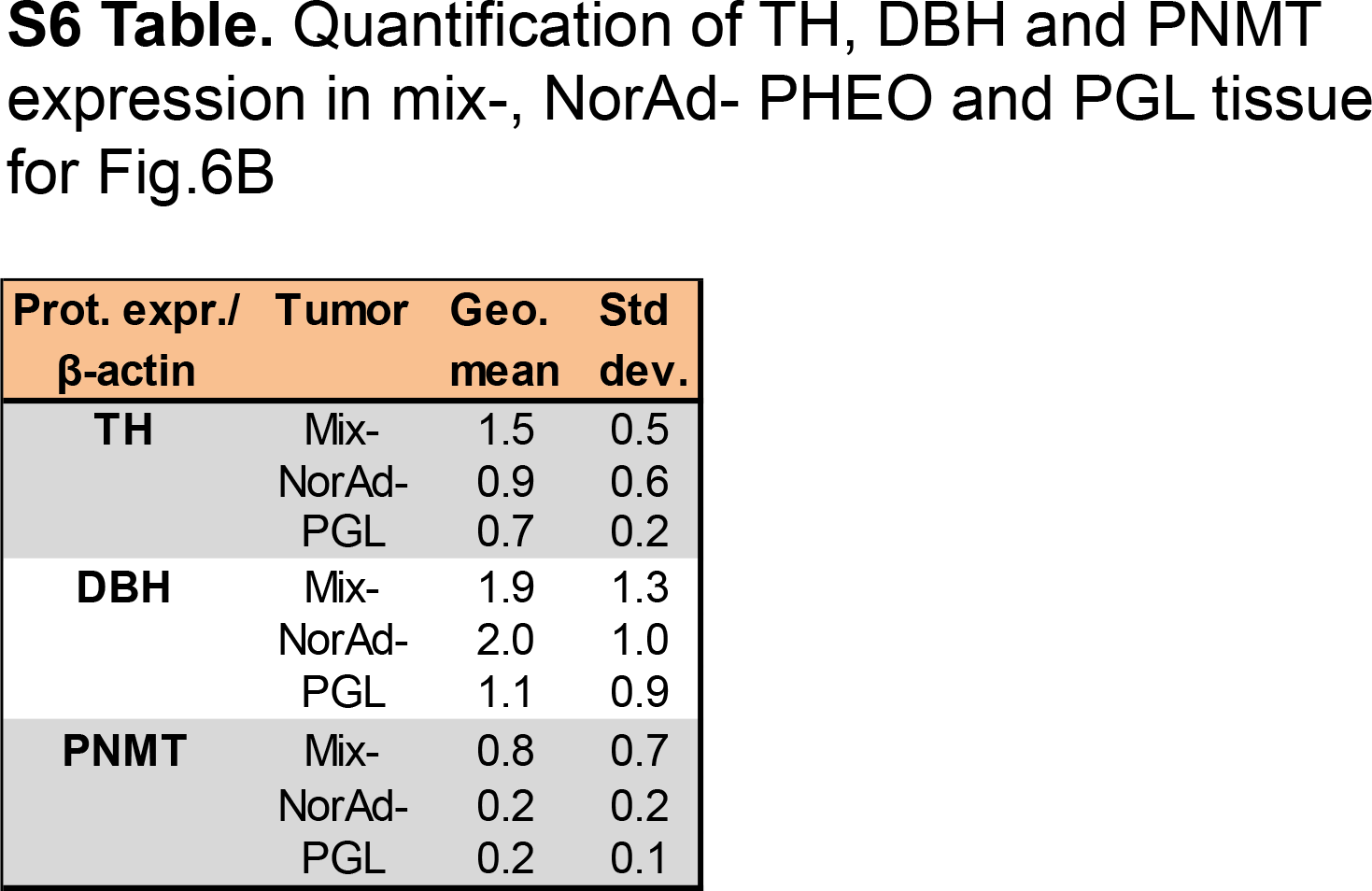

Supplement: S6 Table — Quantification of TH, DBH and PNMT expression in mix-, NorAd- PHEO and PGL tissue. Geographic mean and standard deviation for the values reported. (TIF) [file pone.0125426.s006.tif]

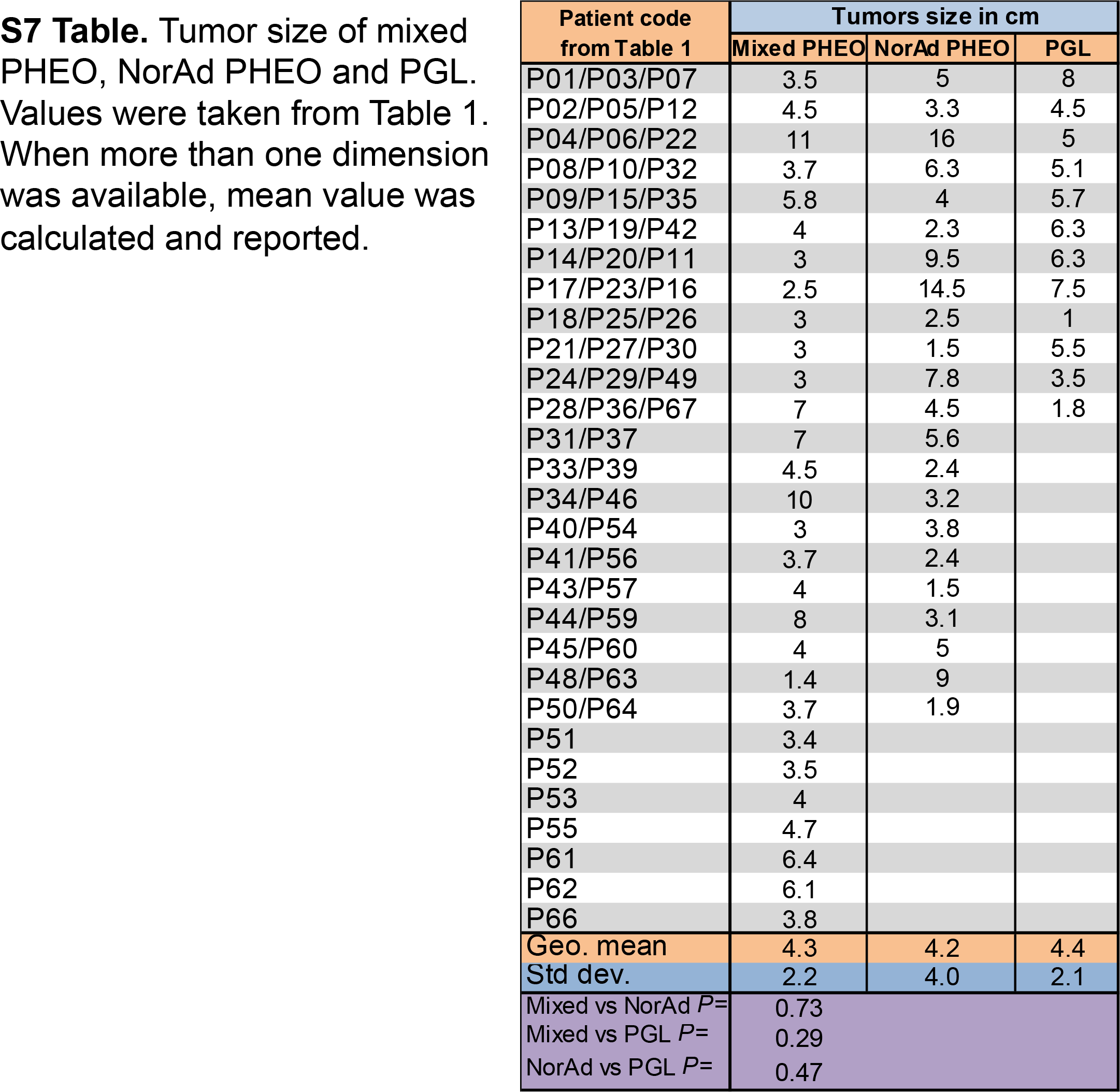

Supplement: S7 Table — Values were taken from Table 1. When more than one dimension was available, mean value was calculated and reported. No significant differences were observed between the three groups. (TIF) [file pone.0125426.s007.tif]
